# Supplementary material for: Lipid levels after childbirth and association with number of children: A population-based cohort study
Source: PLoS One. 2019 Oct 24;14(10):e0223602. doi: 10.1371/journal.pone.0223602 (PMC6812782; doi:10.1371/journal.pone.0223602)
Supplement: S1 Table — Estimates were obtained by logistic regression and adjusted for age at examination, year of first birth, body mass index (linear term), oral contraceptive use, smoking, educational level and time since last meal. (PDF) [file pone.0223602.s001.pdf]

Supplemental Table S1. Crude and adjusted odds ratio (OR) with 95% confidence interval (CI) for one lifetime pregnancy by lipid quintiles in 32 618 parous Norwegian women (≤69 years of age), Cohort of Norway, 1994-2003. Estimates were obtained by logistic regression and adjusted for age at examination, year of the first birth, body mass index (linear term), oral contraceptive use, smoking, educational level and time since last meal.

Lipid quintiles (mmol/l)	1 child mothers (%)	≥ 2 children mothers	total mothers	Crude OR (95%CI) for one lifetime pregnancy	Adjusted OR (95%CI) for one lifetime pregnancy	P[a]
LDL cholesterol						
≤ 2.87	880 (12.4)	6224	7104	1.0 reference	1.0 reference	
2.88-3.38	925 (13.5)	5892	6817	1.11 (1.01-1.23)	1.06 (0.95-1.18)	
3.39-3.89	915 (13.4)	5921	6836	1.09 (0.99-1.21)	1.07 (0.96-1.19)	<0.001
3.90-4.56	876 (13.7)	5493	6369	1.13 (1.02-1.25)	1.10 (0.98-1.24)	
≥ 4.57	894 (16.3)	4598	5492	1.37 (1.24-1.52)	1.30 (1.14-1.45)	
Total cholesterol						
≤ 4.60	840 (11.8)	6258	7098	1.0 reference	1.0 reference	
4.61-5.14	906 (13.0)	6051	6957	1.11 (1.01-1.23)	1.14 (1.02-1.27)	
5.15-5.69	984 (14.2)	5958	6942	1.23 (1.11-1.36)	1.25 (1.12-1.39)	
5.70-6.39	896 (14.3)	5369	6265	1.24 (1.12-1.37)	1.28 (1.15-1.44)	<0.001
≥ 6.40	864 (16.1)	4492	5356	1.43 (1.29-1.59)	1.43 (1.27-1.61)	
TG (Triglyceride)						
≤ 0.74	955 (13.9)	5921	6876	1.0 reference	1.0 reference	
0.75-0.98	907 (13.3)	5929	6836	0.95 (0.86-1.05)	0.93 (0.83-1.03)	
0.99-1.27	889 (13.6)	5637	6526	0.98 (0.88-1.08)	0.93 (0.83-1.03)	0.08
1.28-1.76	855 (13.3)	5568	6423	0.95 (0.86-1.05)	0.88 (0.79-0.99)	
≥ 1.77	884 (14.8)	5073	5957	1.08 (0.98-1.19)	1.01 (0.90-1.14)	
HDL cholesterol						
≤ 1.19	896 (13.5)	5724	6620	0.91 (0.83-1.01)	0.87 (0.78-0.96)	
1.20-1.38	860 (13.1)	5681	6541	0.81 (0.73-0.89)	0.77 (0.69-0.86)	
1.39-1.55	827 (12.7)	5666	6493	0.84 (0.76-0.93)	0.74 (0.66-0.83)	<0.001
1.56-1.79	890 (14.1)	5407	6297	0.87 (0.79-0.96)	0.73 (0.65-0.82)	
≥ 1.80	1017 (15.2)	5650	6667	1.0 reference	1.0 reference	
TG/HDL-c ratio						
≤ 0.45	1002 (14.3)	5978	6980	1.0 reference	1.0 reference	
0.46-0.64	898 (12.8)	5583	6481	0.96 (0.87-1.06)	0.89 (0.79-0.98)	
0.65-0.90	851 (12.9)	5723	6574	0.89 (0.80-0.98)	0.84 (0.75-0.94)	
0.91-1.37	890 (13.6)	5628	6518	0.94 (0.86-1.04)	0.83 (0.74-0.93)	0.01
≥ 1.38	849 (13.9)	5216	6065	0.97 (0.88-1.10)	0.85 (0.76-0.96)	

[a]p for differences between categories (quintiles)
