# Supplementary material for: Lipid levels after childbirth and association with number of children: A population-based cohort study
Source: PLoS One. 2019 Oct 24;14(10):e0223602. doi: 10.1371/journal.pone.0223602 (PMC6812782; doi:10.1371/journal.pone.0223602)
Supplement: S2 Table — Estimates were obtained by logistic regression and adjusted for age at examination, year of first birth, body mass index (linear term), oral contraceptive use, smoking, educational level and time since last meal. (PDF) [file pone.0223602.s002.pdf]

Supplemental Table S2. Adjusted odds ratio (OR) with 95% confidence interval (CI) for one lifetime pregnancy by non-HDL cholesterol quintiles in 32 618 parous Norwegian women (≤69 years of age), Cohort of Norway, 1994-2003. Estimates were obtained by logistic regression and adjusted for age at examination, year of the first birth, body mass index (linear term), oral contraceptive use, smoking, educational level and time since last meal.

Lipid quintiles (mmol/l)	1 child mothers (%)	≥ 2 children mothers	total mothers	OR (95%CI)
Non-HDL cholesterol[a]				
≤ 3.01	817 (12.5)	5716	6533	1.0 reference
3.02-3.54	855 (13.4)	5545	6400	1.03 (0.92-1.16)
3.55-4.06	869 (13.3)	5647	6516	1.05 (0.94-1.17)
4.07-4.73	887 (13.6)	5620	6507	1.08 (0.96-1.22)
≥ 4.74	1040 (15.9)	5488	6528	1.25 (1.10-1.40)

[a] missing cases 134 (0.4%)
