# Supplementary material for: Lipid levels after childbirth and association with number of children: A population-based cohort study
Source: PLoS One. 2019 Oct 24;14(10):e0223602. doi: 10.1371/journal.pone.0223602 (PMC6812782; doi:10.1371/journal.pone.0223602)
Supplement: S3 Table — Estimates were obtained by logistic regression and adjusted for age at examination, year of first birth, body mass index (linear term), oral contraceptive use, smoking, educational level and time since last meal. (PDF) [file pone.0223602.s003.pdf]

Supplemental Table S3. Adjusted odds ratios (ORs) with 95% confidence interval (CI) for one lifetime pregnancy by lipid quintiles in 19 744 parous Norwegian women without reported cardiovascular disease in parents or siblings, Cohort of Norway, 1994-2003. Estimates were obtained by logistic regression and adjusted for age at examination, year of the first birth, body mass index (linear term), oral contraceptive use, smoking, educational level and time since last meal.

Lipid quintiles (mmol/l)	1 child mothers (%)	≥ 2 children mothers	total mothers	OR (95%CI)
LDL cholesterol				
≤ 2.87	569 (11.6)	4327	4896	1.0 reference
2.88-3.38	592 (13.4)	3808	4400	1.11 (0.97-1.27)
3.39-3.89	540 (13.1)	3580	4120	1.06 (0.92-1.22)
3.90-4.56	465 (13.1)	3079	3544	1.08 (0.93-1.25)
≥ 4.57	448 (16.1)	2336	2784	1.30 (1.12-1.53)
Total cholesterol				
≤ 4.60	554 (11.3)	4349	4903	1.0 reference
4.61-5.14	568 (12.8)	3867	4435	1.17 (1.02-1.34)
5.15-5.69	580 (13.9)	3582	4162	1.24 (1.08-1.42)
5.70-6.39	477 (13.5)	3050	3527	1.22 (1.05-1.42)
≥ 6.40	435 (16.0)	2282	2717	1.43 (1.22-1.67)
TG (Triglyceride)				
≤ 0.74	582 (13.0)	3903	4485	1.0 reference
0.75-0.98	542 (12.6)	3741	4283	0.95 (0.83-1.09)
0.99-1.27	515 (13.1)	3406	3921	0.92 (0.82-1.07)
1.28-1.76	476 (12.8)	3229	3705	0.93 (0.80-1.07)
≥ 1.77	499 (14.9)	2851	3350	1.09 (0.93-1.27)
HDL cholesterol				
≤ 1.19	495 (12.8)	3375	3870	0.84 (0.73-0.96)
1.20-1.38	520 (13.4)	3358	3878	0.82 (0.71-0.95)
1.39-1.55	503 (12.6)	3487	3990	0.82 (0.71-0.95)
1.56-1.79	497 (12.8)	3377	3874	0.72 (0.62-0.84)
≥ 1.80	599 (14.5)	3533	4132	1.0 reference
TG/HDL-c ratio				
≤ 0.45	607 (13.5)	3870	4477	1.0 reference
0.46-0.64	542 (13.2)	3547	4089	0.91 (0.78-1.03)
0.65-0.90	473 (11.9)	3490	3963	0.82 (0.71-0.95)
0.91-1.37	522 (13.6)	3301	3823	0.88 (0.77-1.02)
≥ 1.38	470 (13.8)	2922	3392	0.89 (0.76-1.04)
